# Effect of Etomidate Versus Midazolam-Sodium Thiopental on Attenuating the Cardiovascular Response to Laryngoscopy and Tracheal Intubation

**DOI:** 10.5812/aapm-143382

**Published:** 2024-03-14

**Authors:** Hamidreza Shetabi, Darioush Moradi Farsani, Zahra Allafchian

**Affiliations:** 1 Anesthesiology and Critical Care Research Center, Isfahan University of Medical Sciences, Isfahan, Iran; 2 Student Research Committee, Isfahan University of Medical Sciences, Isfahan, Iran

**Keywords:** Cardiovascular, Etomidate, Laryngoscopy, Midazolam, Sodium Thiopental

## Abstract

**Background:**

Laryngoscopy and tracheal intubation lead to an increased sympathetic reflex response, which is associated with increased heart rate and blood pressure. This response can be detrimental in patients with myocardial ischemia. This study aimed to investigate the effects of etomidate in comparison to a combination of midazolam and sodium thiopental in reducing the sympathetic response to laryngoscopy and tracheal intubation.

**Methods:**

This double-blind, randomized clinical trial study was conducted on two groups of 39 candidates for elective surgery under general anesthesia. Anesthesia was induced by etomidate (E) 0.3 mg/kg in the first group and sodium thiopental 2.5 mg/kg and midazolam 0.075 mg/kg in the second group (TM); then, the patients were intubated. Laryngoscopy findings and cardiovascular response were evaluated during the study. Finally, the data were analyzed using SPSS version 23 (IBM SPSS, Armonk, NY, USA).

**Results:**

There was no significant difference between the two groups in terms of age (P = 0.82), weight (P = 0.42), height (P = 0.201), body mass index (P = 0.78), gender (P = 0.65), American Society of Anesthesiologists (ASA) physical status (P = 0.36), and laryngoscopy view grading (P = 0.83). The average laryngoscopy time in the E group was less than the TM group (P = 0.019). In the TM group, at 10 minutes after intubation, mean diastolic blood pressure (P = 0.029) and mean arterial blood pressure (P = 0.023) were significantly lower; however, at other times, there was no significant difference between the two groups (P > 0.05). There was no significant difference between the two groups in terms of adverse responses to laryngoscopy and intubation (P = 0.19).

**Conclusions:**

The results of the present study showed that etomidate (E) and a combination of midazolam-sodium thiopental (TM) acted similarly in attenuating the cardiovascular response to laryngoscopy and tracheal intubation, and it seems that TM can be used instead of E if needed.

## 1. Background

In general anesthesia, laryngoscopy and endotracheal intubation are used to establish a safe airway and prevent pulmonary aspiration (1, 2). Laryngoscopy and endotracheal intubation can cause tachycardia, an increase in blood pressure, and irregular heartbeat, which can cause myocardial ischemia and increase intracranial pressure (ICP) in susceptible individuals (3, 4). These serious complications can be a threat to patients with heart and cerebrovascular diseases (5). The stress response to the laryngoscope occurs through the central nervous system and the sympathetic reflex (6). It is believed that tension in the tissues of the larynx and pharynx during laryngoscopy is the main cause of the sympathetic response (7).

An important factor determining the severity of these hemodynamic disorders is the depth of anesthesia at this stage (8). Hemodynamic changes during intubation and protecting the patient against these severe changes are very important. To date, several drugs have been used to reduce the response to tracheal intubation and reduce the hemodynamic response, including narcotic drugs, beta receptor blockers, and arterial dilators, which can have side effects (9, 10). Etomidate is a hypnotic substance with minimal effects on the cardiovascular system, which is not associated with the release of histamine. Etomidate does not have analgesic properties. Etomidate side effects are injection pain, myoclonus, superficial thrombophlebitis, and a high prevalence of nausea and vomiting.(11) Previous studies have also reported that etomidate does not effectively prevent the sympathetic response to laryngoscopy and tracheal intubation (12, 13).

Thiopental sodium is a useful intravenous anesthetic and is the standard against which recently introduced anesthetics have been compared. However, there are certain limitations to the use of thiopental, such as its long half-life and lack of hemodynamic and sympathetic nervous system response to laryngoscopy and intubation. Inserting the chip.(14) Midazolam is a short-acting benzodiazepine in adults with an elimination half-life of 1.5 to 2.5 hours (15). The therapeutic effects and side effects of midazolam are due to its effects on GABA receptors. In elderly individuals, children, and teenagers, its elimination half-life is longer (16, 17). Considering the importance of hemodynamic stability in response to induction of anesthesia, laryngoscopy, and tracheal intubation, and to the best of the knowledge of the authors, no study has been conducted comparing the effect of etomidate and thiopental sodium-midazolam combination on cardiovascular response to tracheal intubation. Therefore, it was decided to conduct the present study.

## 2. Methods

### 2.1. Patients

This double-blind randomized clinical trial study was conducted on 78 patients at Alzahra hospital, affiliated with Isfahan University of Medical Sciences, Isfahan, Iran, within April 21 2020 to January 4, 2020.

### 2.2. Ethical Considerations

The present study was approved by the Ethics Committee of Isfahan University of Medical Sciences by the code IR.MUI.MED.REC.1398.622, registered with ID number IRCT20180416039326N11 on 2019/14/03 in the Iranian Clinical Trial Center (https://en.irct.ir/user/trial/45573/view). Additionally, consent was also obtained. 

### 2.3. Inclusion and Exclusion Criteria

Candidates for elective surgery (maximum 2 hours) under general anesthesia, aged 18 to 70 years, with physical status 1 and 2 according to the American Society of Anesthesiologists (ASA), and informed consent were included in the study. Obese patients, pregnant women, patients with uncontrolled diabetes, uncontrolled chronic obstructive pulmonary disease (COPD), uncontrolled cardiovascular patients, any uncontrolled systemic disease, chronic use of painkillers or sedatives, body mass index higher than 25 kg/m^2^, predicting the difficulty of tracheal intubation, and history of sensitivity and allergy to the anesthetic drugs used were not included in the study. More than one attempt for tracheal intubation, laryngoscopy duration of more than 15 seconds, and occurrence of severe allergic reactions to stud drugs were considered the exclusion criteria.

### 2.4. Randomization 

Using a computer-based algorithm that followed a random number generator technique, 78 patients were randomly divided into two groups of 39. The participants were categorized using an online calculator at www.calculator. Then, each patient was randomly allocated a number depending on the calculator's output. Numbers 1 to 39 were in the etomidate (E) group; nevertheless, numbers 40 to 78 were in the thiopental sodium-midazolam (TM) group.

### 2.5. Blinding Method

Patients and observers who collected the data were unaware of the type of studied pharmacological intervention. Study drugs were prepared and administered by an anesthesiologist who was not a member of the research team.

### 2.6. Groups and Interventions

Upon entering the operating room, all patients underwent standard monitoring, including electrocardiogram, non-invasive intermittent sphygmomanometer, pulse oximetry, and capnography. After receiving ringer lactate 5 ml/kg, fentanyl 2 mcg/kg was injected in both groups; then, in the E group, etomidate 0.3 mg/kg, and in the TM group, sodium thiopental 2.5 mg/kg and midazolam 0.075 mg/kg (18), were injected. Both groups received atracurium 0.5 mg/kg and were ventilated with a mask and oxygen for 3 minutes. Tracheal intubation was performed using a gender-appropriate tracheal tube (size 7.5 for women and size 8 for men); then, the endotracheal tube cuff was inflated with a pressure of 20 cm H_2_O with a manual manometer to ensure no air leakage. Afterward, the patients underwent mechanical ventilation. Anesthesia was maintained with isoflurane with Mac 0.8 to 1.2 and oxygen- N_2_O at a ratio of 50%. A change of more than 30% in the cardiovascular response (blood pressure and heart rate) to the induction of anesthesia, laryngoscopy, and tracheal intubation was considered a hemodynamic disorder.

### 2.7. Outcomes

Hemodynamic variables, including heart rate, systolic blood pressure (SBP), diastolic blood pressure (DBP), mean arterial pressure (MAP), and peripheral blood oxygen saturation (SpO_2_) at baseline (before the induction of anesthesia), at 1 and 3 minutes after induction, and at 1, 3, 5, and 10 minutes after intubation were measured and recorded. A change of more than 30% in the cardiovascular response (blood pressure and heart rate) to induction of anesthesia. The grading of laryngoscopy, the average duration of laryngoscopy, and the frequency of adverse cardiovascular response during intubation in two groups were also investigated.

### 2.8. Sample Size

A power analysis was conducted based on previous research articles (18, 19) to determine the sample size required for the study. The calculated sample size was 80, with 40 participants assigned to each group. The aim was to achieve a power of 80% and a level of significance of 5% to detect a difference of 20 beats/min or 20 mmHg in paired hemodynamic data.

### 2.9. Statistical Analysis

SPSS software 23 (IBM SPSS Armonk, NY, USA) was used to analyze the data that were collected. To ensure that the data in this analysis were normal, the Shapiro-Wilk test was applied.

All mean comparison tests were carried out as two-tailed tests, with the alpha error of 5% (95% confidence interval [CI]) taken as the upper limit of rejecting or confirming the null hypothesis. The independent *t*-test was used to compare quantitative variables. The Fisher's exact test or the Chi-square test was used to compare qualitative variables. By using repeated measures analysis of variance, changes in quantitative variables over time in each group and between groups were examined.

## 3. Results

**Figure 1.**
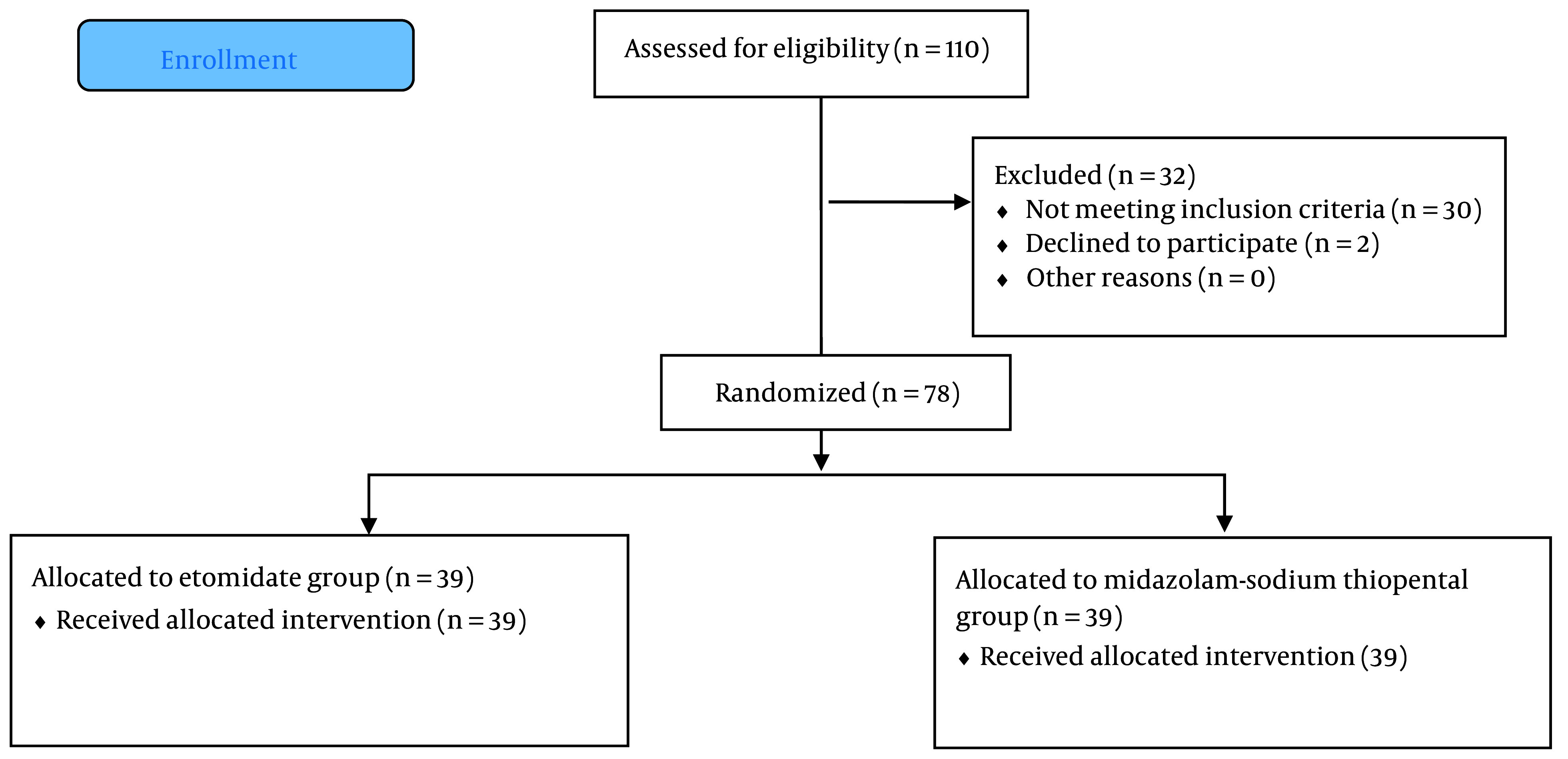
Consort diagram of study

In the present study, out of 80 eligible patients, two participants (one in each group) were excluded due to their refusal to participate in the study. Finally, the analysis was conducted on 78 patients (Figure 1). 

According to Table 1, the two studied groups in terms of demographic and basic variables had no significant difference (P > 0.05).

**Table 1.** Analysis of Demographic Variables in Two Study Groups ^a^

Variables	Etomidate Group (n = 39)	Thiopental-Midazolam Group (n = 39)	P-Value
**Age, y**	45 ± 17.9	45.9 ± 15.8	0.82
**Weight, kg**	66.2 ± 6.3	67.3 ± 6.2	0.42
**Height, cm**	16.8 ± 6.4	169.9 ± 5.9	0.201
**BMI, kg/m** ^**2**^	23.4 ± 1.53	23.30 ± 1.49	0.78
**Gender**			0.65
Male	22 (56.4)	26 (61.5)	
Female	17 (43.6)	15 (38.5)	
**ASA**			0.36
1	16 (41)	20 (51.3)	
2	23 (59)	19 (48.7)	

Abbreviations: BMI, body mass index; ASA, American Society of Anesthesiologists.

^a^ Values are presented as No. (%) or mean ± SD.

According to Table 2, at 10 minutes after intubation, mean DBP (P = 0.029) and MAP (P = 0.023) were significantly lower in the TM group; however, in the rest of the time, there was no significant difference between the two groups.

**Table 2.** Hemodynamic Parameters at the Time Points of the Study in Two Groups

Variables	Time	Etomidate Group (n = 39)	Thiopental-Midazolam Group (n = 39)	P-Value ^a^
**Heart rate (bit per minute)**	T1	81.2 ± 17.3	84.4 ± 16.5	0.40
	T2	81.3 ± 17.2	87.5 ± 16.7	0.066
	T3	82.2 ± 17.2	86.1 ± 12.1	0.24
	T4	89.7 ± 16.7	91.2 ± 18	0.71
	T5	82.7 ± 15.3	85.2 ± 13.7	0.45
	T6	76.9 ± 14.5	81.3 ± 12.3	0.16
	T7	74.3 ± 14.4	80.4 ± 13.8	0.59
	P ^b^	< 0.001	< 0.001	18 ^c^
**SBP (mmHg)**	T1	135.1 ± 17	131 ± 16.3	0.28
	T2	123.5 ± 21.4	120.3 ± 21.3	0.52
	T3	120.9 ± 23.4	118.7 ± 19.4	0.66
	T4	146.5 ± 26.9	135.5 ± 23.8	0.062
	T5	128.8 ± 21.8	132.2 ± 20.3	0.24
	T6	125.6 ± 25.3	116.8 ± 18.5	0.08
	T7	119.9.6 ± 20.8	113.8 ± 17	0.18
	P ^b^	< 0.001	< 0.001	0.12 ^c^
**DBP (mmHg)**	T1	86.7 ± 11.8	82.4 ± 11.9	0.20
	T2	76.1 ± 15.9	75.7 ± 16.9	0.93
	T3	87.7 ± 19.3	75.5 ± 17.9	0.48
	T4	91.9 ± 19.3	84.7 ± 18.5	0.096
	T5	79.3 ± 12.4	76.6 ± 17	0.42
	T6	77 ± 17.3	71.2 ± 15.4	0.12
	T7	78 ± 19.2	69 ± 16.4	0.029
	P ^b^	< 0.001	< 0.001	0.07 ^c^
**MAP (mmHg)**	T1	10.4.3 ± 14.8	100.2 ± 13.7	0.21
	T2	10.4.3 ± 19	921. ± 18.6	0.86
	T3	93.6 ± 22	92.1 ± 18.8	0.75
	T4	112.3 ± 20.2	105.7 ± 21.8	0.17
	T5	97.1 ± 15.5	94.6 ± 18.4	0.52
	T6	95.7 ± 21.9	87.6 ± 17.3	0.074
	T7	93.8 ± 19.5	84.5 ± 15.8	0.023
	P ^b^	< 0.001	0.006	0.20 ^c^
**SpO**_**2**_ ** (%)**	T1	96.5 ± 2.6	96.7 ± 2.8	0.15
	T2	97.7 ± 1.9	97.5 ± 6.5	0.79
	T3	98.4 ± 1.4	97.9 ± 6	0.66
	T4	97.9 ± 2.2	98.3 ± 3.3	0.60
	T5	98.5 ± 1.2	98.2 ± 4.6	0.74
	T6	98.5 ± 1.2	98 ± 5	0.5
	T7	98.4 ± 1.4	97.9 ± 4.9	0.5
	P ^b^	< 0.001	0.24	0.92 ^c^

Abbreviations: SBP, systolic blood pressure; DBP, diastolic blood pressure; MAP, mean arterial pressure; SpO_2_, peripheral blood oxygen saturation.

^a^ Significant level of difference between two groups at each point of time according to the *t*-test.

^b^ Significant level of parameter changes within each group according to variance analysis test with repeated observations.

^c^ Significant level of the process of parameter changes between two groups according to the analysis of variance test with repeated observations.

T1 (baseline); T2, T3 (1 and 2 minutes after the induction of anesthesia); T4, T5, T6, and T7(1, 3, 5 and 10 minutes after intubation).

In the intra-group analysis, there was a significant difference in the changes in the heart rate, SBP, DBP, and MAP in both groups (P < 0.05). Nevertheless, SpO_2_ changes were significant only in the E group (P < 0.001).

From the point of view of laryngoscopy, no significant difference was observed between the two groups (P = 0.83); however, the average duration of laryngoscopy in the E group (10.79 ± 2.66 seconds) was significantly less than in the TM group (12.13 ± 2.22 seconds) (P = 0.019). The first attempt at tracheal intubation for all patients of both groups was successful. There was no significant difference between the two groups in terms of adverse response during intubation (hypertension, tachycardia, cough, and movement) (P = 0.56; Table 3). 

**Table 3.** Laryngoscopy Grading, Average Duration of Laryngoscopy, and Frequency of Cardiovascular Adverse Response During Intubation in Two Groups ^a^

Variables	Etomidate Group (n = 39)	Thiopental-Midazolam Group (n = 39)	P-Value
**Laryngoscopy grade**			0.83
1	21 (53.8)	22 (56.4)	
2	15 (38.5)	12 (30.8)	
3	2 (5.1)	3 (7.7)	
4	1 (2.6)	2 (5.1)	
**Mean laryngoscopy time (sec)**	10.79 ± 2.66	12.13 ± 2.22	0.019
**Adverse response to laryngoscopy**			0. 19
Hypertension	5 (12.8)	1 (2.6)	
Tachycardia	1 (2.6)	0 (0)	
Cough	0 (0)	2 (5.1)	
Movement	3 (7.7)	2 (5.1)	

^a^ Values are presented as No. (%).

## 4. Discussion

The findings of the present study showed that the changes in hemodynamic parameters, including blood pressure, heart rate, and SpO_2_, were similar in the two groups receiving the E and TM combination; however, 10 minutes after intubation, the TM group had lower mean DBP and MAP. Additionally, there were significant changes in blood pressure and heart rate during the study period in both groups. The trend of changes in SpO_2_ was significant in the E group and not significant in the TM group. On the other hand, the frequency of hypertension was higher in the etomidate group; nevertheless, no significant difference was observed between the two groups. The findings of the present study showed that the changes in hemodynamic parameters, including blood pressure, heart rate, and SpO_2_, were similar in the two groups receiving the E and TM combination; nonetheless, 10 minutes after intubation, the TM group had lower mean DBP and MAP.

The frequency of adverse responses to laryngoscopy and tracheal intubation, including tachycardia, increased blood pressure, and irregular heart rate, was not different between the two groups. In this regard, the results of Masoudifar et al.'s study comparing the effects of the induction of anesthesia to three drugs, etomidate, propofol, and sodium thiopental, on hemodynamic status in laryngoscopy suspension surgery, did not report significant differences between the three groups in terms of hemodynamic changes (20). Kim et al. investigated the effect of etomidate and sodium thiopental during the induction of general anesthesia and tracheal intubation on hemodynamic parameters in elderly patients. Based on the results, the hemodynamic changes in the two groups were not significantly different (21).

In the present study, there was no significant difference between the two study groups in terms of cardiovascular response to tracheal intubation, which is similar to the above-mentioned studies (20, 21). Another study conducted by BAŞ et al. investigated the effect of sodium thiopental, etomidate, propofol, and midazolam on the hemodynamic status in cardiac surgery, in which patients receiving midazolam had higher hemodynamic stability, and midazolam was introduced as a safer drug (22).

Sonday et al. investigated thiopental versus etomidate for rapid sequence intubation. As a result, they observed no clinical difference between the use of etomidate or thiopental with succinylcholine for rapid sequence intubation (23). In a study by Habibi et al. on patients who were candidates for elective coronary artery bypass graft (CABG) surgery, either etomidate or ketamine-thiopental sodium was used to induce anesthesia. It was observed that after the induction of anesthesia with the administration of ketamine-thiopental sodium, hemodynamic stability was better than that of sodium thiopental (24).

In another study on patients aged 18 to 45 years who were candidates for elective surgery, they used etomidate (0.3 mg/kg) in the first group, propofol (1.5 mg/kg) and ketamine in the second group, and thiopental (3 mg/kg) and ketamine (0.5 mg/kg) in the third group. The results showed that the propofol-ketamine combination can act as an effective and safe induction agent to reduce hemodynamic responses to laryngoscopy and intubation, along with hemodynamic stability (25).

According to the findings of the present study, the duration of laryngoscopy in the group receiving etomidate was significantly less than that of TM. On the other hand, the complications during laryngoscopy were not significantly different between the two groups, which is consistent with the findings of Masoudifar et al.'s study (20). At the same time, according to the findings of the present study, it seems that the cardiovascular response to the combination of TM is similar to etomidate.

There were certain limitations in the present study, including the small sample size and the study being limited to one center. Furthermore, this study did not utilize invasive blood pressure monitoring, which could have offered a more comprehensive understanding by providing beat-to-beat recordings of the parameters. This decision was made due to financial limitations. Additionally, plasma catecholamine levels were not monitored, which is suggested to be considered in future studies.

### 4.1. Conclusions

The results of the present study showed that etomidate (E) and a combination of midazolam-sodium thiopental (TM) acted similarly in weakening the cardiovascular response to laryngoscopy and tracheal intubation, and it seems that TM can be used instead of E if needed.

## Data Availability

The dataset presented in the study is available on request from the corresponding author during submission or after its publication. The data are not publicly available yet.
